# Burden of Total and Cause-Specific Mortality Related to Tobacco Smoking among Adults Aged ≥45 Years in Asia: A Pooled Analysis of 21 Cohorts

**DOI:** 10.1371/journal.pmed.1001631

**Published:** 2014-04-22

**Authors:** Wei Zheng, Dale F. McLerran, Betsy A. Rolland, Zhenming Fu, Paolo Boffetta, Jiang He, Prakash Chandra Gupta, Kunnambath Ramadas, Shoichiro Tsugane, Fujiko Irie, Akiko Tamakoshi, Yu-Tang Gao, Woon-Puay Koh, Xiao-Ou Shu, Kotaro Ozasa, Yoshikazu Nishino, Ichiro Tsuji, Hideo Tanaka, Chien-Jen Chen, Jian-Min Yuan, Yoon-Ok Ahn, Keun-Young Yoo, Habibul Ahsan, Wen-Harn Pan, You-Lin Qiao, Dongfeng Gu, Mangesh Suryakant Pednekar, Catherine Sauvaget, Norie Sawada, Toshimi Sairenchi, Gong Yang, Renwei Wang, Yong-Bing Xiang, Waka Ohishi, Masako Kakizaki, Takashi Watanabe, Isao Oze, San-Lin You, Yumi Sugawara, Lesley M. Butler, Dong-Hyun Kim, Sue K. Park, Faruque Parvez, Shao-Yuan Chuang, Jin-Hu Fan, Chen-Yang Shen, Yu Chen, Eric J. Grant, Jung Eun Lee, Rashmi Sinha, Keitaro Matsuo, Mark Thornquist, Manami Inoue, Ziding Feng, Daehee Kang, John D. Potter

**Affiliations:** 1 Division of Epidemiology, Department of Medicine, Vanderbilt University, Nashville, Tennessee, United States of America; 2 Vanderbilt Epidemiology Center, Vanderbilt University, Nashville, Tennessee, United States of America; 3 Vanderbilt-Ingram Cancer Center, Vanderbilt University, Nashville, Tennessee, United States of America; 4 Fred Hutchinson Cancer Research Center, Seattle, Washington, United States of America; 5 Department of Radiation and Medical Oncology, Zhongnan Hospital of Wuhan University, Wuhan, China; 6 The Tisch Cancer Institute, Ichan School of Medicine at Mount Sinai, New York, New York, United States of America; 7 International Prevention Research Institute, Lyon, France; 8 Department of Epidemiology, Tulane University School of Public Health and Tropical Medicine, New Orleans, Louisiana, United States of America; 9 Healis-Sekhsaria Institute for Public Health, Navi Mumbai, India; 10 Division of Radiation Oncology, Regional Cancer Center, Medical College Campus, Trivandrum, India; 11 Epidemiology and Prevention Division, Research Center for Cancer Prevention and Screening, National Cancer Center, Tokyo, Japan; 12 Department of Health and Social Services, Ibaraki Prefectural Government, Ibaraki, Japan; 13 Department of Public Health, Aichi Medical University School of Medicine, Aichi, Japan; 14 Department of Epidemiology, Shanghai Cancer Institute, Shanghai, China; 15 Duke–National University of Singapore Graduate Medical School, Singapore; 16 Saw Swee Hock School of Public Health, National University of Singapore, Singapore; 17 Radiation Effects Research Foundation, Hiroshima, Japan; 18 Division of Epidemiology, Miyagi Cancer Center Research Institute, Natori, Japan; 19 Tohoku University Graduate School of Medicine, Sendai, Japan; 20 Division of Epidemiology and Prevention, Aichi Cancer Center Research Institute, Nagoya, Japan; 21 Genomics Research Center, Academia Sinica, Taipei, Taiwan; 22 Graduate Institute of Epidemiology, College of Public Health, National Taiwan University, Taipei, Taiwan; 23 Division of Cancer Control and Population Sciences, University of Pittsburgh Cancer Institute, Pittsburgh, Pennsylvania, United States of America; 24 Department of Epidemiology, Graduate School of Public Health, University of Pittsburgh, Pittsburgh, Pennsylvania, United States of America; 25 Department of Preventive Medicine, Seoul National University College of Medicine, Seoul, Republic of Korea; 26 Department of Health Studies, University of Chicago, Chicago, Illinois, United States of America; 27 Department of Medicine, University of Chicago, Chicago, Illinois, United States of America; 28 Department of Human Genetics, University of Chicago, Chicago, Illinois, United States of America; 29 University of Chicago Cancer Research Center, University of Chicago, Chicago, Illinois, United States of America; 30 Institute of Biomedical Sciences, Academia Sinica, Taipei, Taiwan; 31 Department of Biochemical Science and Technology, National Taiwan University, Taipei, Taiwan; 32 Department of Cancer Epidemiology, Cancer Institute/Hospital, Chinese Academy of Medical Sciences, Beijing, China; 33 Fuwai Hospital and Cardiovascular Institute, Chinese Academy of Medical Sciences, Beijing, China; 34 China National Center for Cardiovascular Disease, Beijing, China; 35 Screening Group, Prevention and Early Detection Section, International Agency for Research on Cancer, Lyon, France; 36 Department of Public Health, Dokkyo Medical University School of Medicine, Tochigi, Japan; 37 Department of Medical Oncology and Immunology, Nagoya City University Graduate School of Medical Science, Nagoya, Japan; 38 Department of Social and Preventive Medicine, Hallym University College of Medicine, Okcheon-dong, Republic of Korea; 39 Department of Preventive Medicine, Seoul National University College of Medicine, Seoul National University, Seoul, Republic of Korea; 40 Department of Environmental Health Sciences, Mailman School of Public Health, Columbia University, New York, New York, United States of America; 41 Division of Preventive Medicine and Health Services Research, Institute of Population Health Sciences, National Health Research Institutes, Miaoli, Taiwan; 42 Taiwan Biobank, Institute of Biomedical Sciences, Academia Sinica, Taipei, Taiwan; 43 Graduate Institute of Environmental Science, China Medical University, Taichung, Taiwan; 44 Department of Environmental Medicine, New York University School of Medicine, New York, New York, United States of America; 45 Department of Food and Nutrition, Sookmyung Women's University, Seoul, Republic of Korea; 46 Nutritional Epidemiology Branch, Division of Cancer Epidemiology and Genetics, National Cancer Institute, National Institutes of Health, Department of Health and Human Services, Rockville, Maryland, United States of America; 47 Graduate School of Medicine, University of Tokyo, Tokyo, Japan; 48 Research Center for Cancer Prevention and Screening, National Cancer Center, Tokyo, Japan; 49 Department of Biostatistics, University of Texas MD Anderson Cancer Center, Houston, Texas, United States of America; San Diego State University, United States of America

## Abstract

**Background:**

Tobacco smoking is a major risk factor for many diseases. We sought to quantify the burden of tobacco-smoking-related deaths in Asia, in parts of which men's smoking prevalence is among the world's highest.

**Methods and Findings:**

We performed pooled analyses of data from 1,049,929 participants in 21 cohorts in Asia to quantify the risks of total and cause-specific mortality associated with tobacco smoking using adjusted hazard ratios and their 95% confidence intervals. We then estimated smoking-related deaths among adults aged ≥45 y in 2004 in Bangladesh, India, mainland China, Japan, Republic of Korea, Singapore, and Taiwan—accounting for ∼71% of Asia's total population. An approximately 1.44-fold (95% CI = 1.37–1.51) and 1.48-fold (1.38–1.58) elevated risk of death from any cause was found in male and female ever-smokers, respectively. In 2004, active tobacco smoking accounted for approximately 15.8% (95% CI = 14.3%–17.2%) and 3.3% (2.6%–4.0%) of deaths, respectively, in men and women aged ≥45 y in the seven countries/regions combined, with a total number of estimated deaths of ∼1,575,500 (95% CI = 1,398,000–1,744,700). Among men, approximately 11.4%, 30.5%, and 19.8% of deaths due to cardiovascular diseases, cancer, and respiratory diseases, respectively, were attributable to tobacco smoking. Corresponding proportions for East Asian women were 3.7%, 4.6%, and 1.7%, respectively. The strongest association with tobacco smoking was found for lung cancer: a 3- to 4-fold elevated risk, accounting for 60.5% and 16.7% of lung cancer deaths, respectively, in Asian men and East Asian women aged ≥45 y.

**Conclusions:**

Tobacco smoking is associated with a substantially elevated risk of mortality, accounting for approximately 2 million deaths in adults aged ≥45 y throughout Asia in 2004. It is likely that smoking-related deaths in Asia will continue to rise over the next few decades if no effective smoking control programs are implemented.

*Please see later in the article for the Editors' Summary*

## Abstract 2

Wei Zheng and colleagues quantify the burden of tobacco-smoking-related deaths for adults in Asia.

*Please see later in the article for the Editors' Summary*

## Editors' Summary

**Background:**

Every year, more than 5 million smokers die from tobacco-related diseases. Tobacco smoking is a major risk factor for cardiovascular disease (conditions that affect the heart and the circulation), respiratory disease (conditions that affect breathing), lung cancer, and several other types of cancer. All told, tobacco smoking kills up to half its users. The ongoing global “epidemic” of tobacco smoking and tobacco-related diseases initially affected people living in the US and other Western countries, where the prevalence of smoking (the proportion of the population that smokes) in men began to rise in the early 1900s, peaking in the 1960s. A similar epidemic occurred in women about 40 years later. Smoking-related deaths began to increase in the second half of the 20th century, and by the 1990s, tobacco smoking accounted for a third of all deaths and about half of cancer deaths among men in the US and other Western countries. More recently, increased awareness of the risks of smoking and the introduction of various tobacco control measures has led to a steady decline in tobacco use and in smoking-related diseases in many developed countries.

**Why Was This Study Done?:**

Unfortunately, less well-developed tobacco control programs, inadequate public awareness of smoking risks, and tobacco company marketing have recently led to sharp increases in the prevalence of smoking in many low- and middle-income countries, particularly in Asia. More than 50% of men in many Asian countries are now smokers, about twice the prevalence in many Western countries, and more women in some Asian countries are smoking than previously. More than half of the world's billion smokers now live in Asia. However, little is known about the burden of tobacco-related mortality (deaths) in this region. In this study, the researchers quantify the risk of total and cause-specific mortality associated with tobacco use among adults aged 45 years or older by undertaking a pooled statistical analysis of data collected from 21 Asian cohorts (groups) about their smoking history and health.

**What Did the Researchers Do and Find?:**

For their study, the researchers used data from more than 1 million participants enrolled in studies undertaken in Bangladesh, India, mainland China, Japan, the Republic of Korea, Singapore, and Taiwan (which together account for 71% of Asia's total population). Smoking prevalences among male and female participants were 65.1% and 7.1%, respectively. Compared with never-smokers, ever-smokers had a higher risk of death from any cause in pooled analyses of all the cohorts (adjusted hazard ratios [HRs] of 1.44 and 1.48 for men and women, respectively; an adjusted HR indicates how often an event occurs in one group compared to another group after adjustment for other characteristics that affect an individual's risk of the event). Compared with never smoking, ever smoking was associated with a higher risk of death due to cardiovascular disease, cancer (particularly lung cancer), and respiratory disease among Asian men and among East Asian women. Moreover, the researchers estimate that, in the countries included in this study, tobacco smoking accounted for 15.8% of all deaths among men and 3.3% of deaths among women in 2004—a total of about 1.5 million deaths, which scales up to 2 million deaths for the population of the whole of Asia. Notably, in 2004, tobacco smoking accounted for 60.5% of lung-cancer deaths among Asian men and 16.7% of lung-cancer deaths among East Asian women.

**What Do These Findings Mean?:**

These findings provide strong evidence that tobacco smoking is associated with a substantially raised risk of death among adults aged 45 years or older throughout Asia. The association between smoking and mortality risk in Asia reported here is weaker than that previously reported for Western countries, possibly because widespread tobacco smoking started several decades later in most Asian countries than in Europe and North America and the deleterious effects of smoking take some years to become evident. The researchers note that certain limitations of their analysis are likely to affect the accuracy of its findings. For example, because no data were available to estimate the impact of secondhand smoke, the estimate of deaths attributable to smoking is likely to be an underestimate. However, the finding that nearly 45% of the global deaths from active tobacco smoking occur in Asia highlights the urgent need to implement comprehensive tobacco control programs in Asia to reduce the burden of tobacco-related disease.

**Additional Information:**

Please access these websites via the online version of this summary at http://dx.doi.org/10.1371/journal.pmed.1001631.

- The World Health Organization provides information about the dangers of tobacco (in several languages) and about the WHO Framework Convention on Tobacco Control, an international instrument for tobacco control that came into force in February 2005 and requires parties to implement a set of core tobacco control provisions including legislation to ban tobacco advertising and to increase tobacco taxes; its 2013 report on the global tobacco epidemic is available
- The US Centers for Disease Control and Prevention provides detailed information about all aspects of smoking and tobacco use
- The UK National Health Services Choices website provides information about the health risks associated with smoking
- MedlinePlus has links to further information about the dangers of smoking (in English and Spanish)
- SmokeFree, a website provided by the UK National Health Service, offers advice on quitting smoking and includes personal stories from people who have stopped smoking
- Smokefree.gov, from the US National Cancer Institute, offers online tools and resources to help people quit smoking

## Introduction

Tobacco smoking is a major risk factor for many diseases, including cardiovascular disease (CVD), respiratory disease, and cancers of the lung and multiple other sites [1],[2]. In the US and many other Western countries, the epidemic of tobacco smoking started in men in the early 1900s and reached its peak in the 1960s; a similar epidemic occurred among women ∼40 y later [3]–[5]. The main increase in tobacco-related deaths in these countries was not seen until the second half of the 20th century [3],[6]–[8]. By the 1990s, tobacco smoking accounted for an estimated one-third of all deaths and >50% of cancer deaths in adult men [3],[6]–[8]. With increasing awareness of smoking-associated risks and heightened anti-smoking campaigns, tobacco use has steadily declined in the US and many other developed countries over the past 20–30 y [3]–[5],[9],[10], resulting in a recent decrease in lung cancer and other smoking-related diseases in these countries [3],[11].

In Asia, where ∼60% of the world population lives, tobacco control programs are less well developed, particularly in low- and middle-income countries including China and India, the two most populous countries in the world. Inadequate public awareness of smoking risks, combined with aggressive marketing by tobacco companies, has resulted in a sharp increase in tobacco smoking among men in many Asian countries over the past few decades [3],[11],[12]. Smoking prevalence in women was traditionally very low but has increased in recent decades in some Asian countries [3],[11],[12]. More than 50% of men in many Asian countries are smokers [12],[13], approximately twice the level in many Western countries. Despite a recent decline in smoking prevalence in several high-income Asian countries [11],[13], tobacco use in most Asian countries remains very high. Indeed, Asia is now considered the largest tobacco producer and consumer in the world. More than half of the world's 1.1 billion smokers live in Asia [3],[13]. Because many Asian countries are in the early stages of the tobacco epidemic, it is likely that the burden of diseases caused by tobacco smoking will continue to rise over the next few decades, and much longer if the tobacco epidemic remains unchecked.

The size of the effect of tobacco smoking on risk of death, typically measured using smoking-associated relative risks (RRs), varies across countries because of differences in characteristics of smokers, smoking behaviors, and tobacco products. Over the past 15 y, several studies have investigated associations between smoking and selected health outcomes in certain Asian populations and have estimated smoking-associated population attributable risk (PAR) [14]–[21]. Some studies estimated burden of disease due to smoking in a specific Asian country/region [14],[16],[17],[19],[20]. However, most of these estimates were derived from either a single cohort study or studies using a less-than-optimal research design. In this study, we first estimated RRs of overall and cause-specific mortality associated with tobacco smoking as well as smoking prevalence, using data from ∼1 million participants recruited in 21 prospective cohort studies in seven countries/regions that account for ∼71% of Asia's total population. We then used these estimates and mortality data from the World Health Organization [22] to quantify deaths attributable to tobacco smoking in these Asian populations.

## Methods

This study was approved by the ethics committees for all the participating studies and of the Fred Hutchinson Cancer Research Center.

This study utilized resources from a recent pooling project of prospective cohort studies conducted as part of the Asia Cohort Consortium that quantified the association between body mass index and risk of overall and cause-specific mortality in Asians [23]. Cohorts included in the current analysis were in Bangladesh, India, mainland China, Japan, Republic of Korea, Singapore, and Taiwan. A brief description of each of the participating cohort studies is provided in Text S1. All of the cohort studies collected baseline data on demographics, lifestyle factors, body mass index, and history of tobacco smoking, which included current smoking status, duration, and amount and types of tobacco products. Data on all-cause and cause-specific mortality were ascertained through linkage to death certificate data or active follow-up. Additional data were collected on other baseline variables, including education, marital status, alcohol consumption, physical activity, and previous diagnosis of selected diseases, including diabetes, hypertension, cancer, and CVDs. Individual-level data from all participating cohorts were collected and harmonized for statistical analysis.

The association between tobacco smoking and risk of death was examined using Cox proportional hazards regression models, employing a categorical representation of tobacco smoking as the predictor variable. Lifetime nonsmokers were used as the reference for estimating hazard ratios (HRs)—as measures of RR of death for the exposed versus the non-exposed population—and 95% confidence intervals associated with ever, former, and current smoking, as well as pack-years smoked, after adjusting for potential confounders including baseline age, education, urban/rural residence, body mass index, and marital status. All analyses were conducted separately for men and women because of large differences in smoking prevalence. Analyses were country-specific unless otherwise noted. To improve the stability of point estimates in the analyses of pack-years of smoking and for risk of death due to site-specific cancer, as well as types of CVD and respiratory diseases, cohorts were combined into broad ethnic groupings: South Asians (Indians and Bangladeshis) and East Asians (Chinese [including cohorts from mainland China, Singapore, and Taiwan], Japanese, and Koreans), and categorized further among East Asians into Chinese/Koreans and Japanese. No smoking-associated HR was estimated for Bangladesh separately because of the small sample size. The number of Koreans in this study was small, and, thus, they were combined with Chinese individuals in some analyses. Bidi smoking is common in India and Bangladesh; thus, information regarding bidi smoking was incorporated to construct smoking variables, including pack-years smoked (4 bidis = 1 cigarette based on approximately 0.25 and 1.0 g of tobacco per bidi and cigarette, respectively).

In the models, the effect of tobacco smoking on mortality was assumed to be cohort-specific. For each cohort, we assumed that the log-HR for tobacco smoking has a fixed-effect component that is common to all cohorts within each country and a random effect that is cohort-specific. Random effects for log-HRs were assumed to be normally distributed, with mean zero; that is, we assumed that 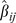, the estimated log-HR for the *j*-th smoking level in the *i*-th cohort, has distribution 
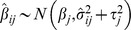
, where 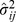 is the within-study variance of 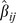 as estimated from the Cox regression model and 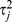 is the between-cohort variance of 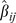 [24],[25]. Parameter *β_j_* and 95% CIs were estimated in the meta-analysis. Age at study entry and exit was used to define the time-to-event variable in the Cox models. Age at study exit was defined as age at date of death or end of follow-up, whichever occurred first. Cox model estimation for each cohort was performed using the PHREG procedure in SAS version 9.2. Meta-analysis estimation was performed using the SAS MIXED procedure.

To estimate PAR, we used the following formula: PAR = P(RR−1)/[P(RR−1)+1], where smoking prevalence and smoking-associated RR are denoted as P and RR (measured using HR in this analysis), respectively. PARs for overall mortality and major causes of death associated with tobacco smoking were estimated for each cohort and then combined using meta-analyses to derive summary PARs per country. To estimate PARs for East Asians (Chinese, Japanese, and Koreans), South Asians (Bangladeshis and Indians), or all seven countries/regions combined, we used the population size of each country/region as a weight to derive weighted HR and smoking prevalence values. To estimate the number of deaths attributable to tobacco smoking, we used World Health Organization age-specific death rates for 2004 for each country. Most of the cohort studies enrolled participants after the mid-1980s; therefore, smoking prevalence rates estimated in this study reflect smoking status in the 1990s (Table 1). Given the long latency of chronic diseases—typically 15 y and longer—it is reasonable to use smoking prevalence rates assessed in the 1990s to estimate number of deaths due to tobacco smoking in 2004.

**Table 1:** Characteristics of participating cohorts in the Asia Cohort Consortium.

Cohort	Number of Participants^a^	Study Entry	Mean Years of Follow-Up	Women (Percent)	Mean Age at Entry	Ever-Smokers (Percent)		Number of Deaths	Cause of Death (Percent)^b^			
						Men	Women		Cancer	CVD	Respiratory Diseases	Other
**India**												
Mumbai	120,055	1991–1997	5.3	36.4	53.4	31.8	0.5	10,839	8.5	45.0	14.4	32.2
Trivandrum	103,942	1995–2002	7.8	59.6	52.7	60.1	1.8	9,406	10.6	36.6	12.8	40.0
Bangladesh	4,572	2000–2002	6.7	41.0	46.8	83.0	15.5	206	13.7	51.2	10.2	24.9
**Mainland China**												
CHEFS	137,460	1990–1992	7.8	50.9	54.9	63.9	13.4	14,776	23.4	44.8	5.0	26.8
SCS	18,010	1986–1989	16.4	0.0	55.2	57.2	NA	4,902	39.6	33.9	10.7	15.9
SMHS	54,707	2001–2006	3.1	0.0	55.1	69.6	NA	596	53.1	25.7	5.4	15.7
SWHS	67,245	1996–2000	8.7	100.0	51.3	NA	2.7	1,921	48.2	23.5	2.6	25.7
**Taiwan**												
CBCSP	22,961	1991–1992	15.4	50.1	47.2	56.4	1.0	2,400	38.1	19.5	5.9	36.4
CVDFACTS	4,170	1990–1993	15.0	55.8	50.7	54.9	1.3	711	27.5	26.1	10.7	35.7
Singapore (SCHS)	57,714	1993–1999	11.7	56.1	56.1	57.1	8.4	8,234	36.7	33.1	14.8	15.4
**Japan**												
3 Pref Aichi	29,316	1985	12.1	50.6	56.3	84.3	17.5	5,330	32.4	35.0	11.9	20.7
Ibaraki	91,847	1993–1994	11.6	66.3	58.5	77.8	5.6	9,545	NA	NA	NA	NA
JACC	74,465	1988–1990	12.9	56.4	57.0	79.1	6.6	10,099	38.6	29.1	11.4	20.9
JPHC1	40,574	1990–1992	14.7	52.2	49.6	75.7	7.3	3,007	45.0	24.6	6.0	24.3
JPHC2	52,838	1992–1995	11.7	52.9	54.1	75.7	7.6	4,708	44.6	24.1	8.7	22.6
3 Pref Miyagi	18,951	1984	12.0	53.4	56.2	77.1	12.0	3,307	31.0	38.5	11.0	19.5
Miyagi	38,560	1990	12.9	45.2	51.5	81.5	11.1	2,932	54.9	25.9	6.3	12.9
Ohsaki	37,884	1995	10.5	47.0	59.5	81.1	11.0	5,093	37.4	30.7	12.9	19.0
RERF	47,532	1963–1993	22.0	59.2	51.6	86.2	15.5	24,128	27.4	37.2	13.3	22.2
**Republic of Korea**												
KMCC	13,446	1993–2004	6.6	62.5	57.9	79.1	10.0	1,036	29.3	24.8	8.6	37.3
Seoul	13,680	1992–1993	14.7	0.0	49.2	77.3	NA	799	53.6	16.8	3.0	26.7
**Total**	1,049,929	1963–2006	10.2	51.4	54.3	65.1	7.1	123,975	29.8	35.0	10.8	24.3

a Including only participants eligible for the current analysis.

b Deaths from unknown causes are not included.

3 Pref, Three Prefecture Cohort Study; CBCSP, Community-Based Cancer Screening Project; CHEFS, China National Hypertension Survey Epidemiology Follow-Up Study; CVDFACTS, Cardiovascular Disease Risk Factor Two-Township Study; JACC, Japan Collaborative Cohort Study, JPHC, Japan Public Health Center-Based Prospective Study; KMCC, Korea Multi-Center Cancer Cohort; NA, not available; RERF, Radiation Effects Research Foundation; SCHS, Singapore Chinese Health Study; SCS, Shanghai Cohort Study; SMHS, Shanghai Men's Health Study; SWHS, Shanghai Women's Health Study.

The number of deaths from a particular disease attributable to tobacco smoking was calculated by multiplying the PAR for that disease by the total number of deaths in the population from that disease. Analyses also were performed to estimate the number of deaths from a particular disease due to smoking for age groups 45–59, 60–69, and ≥70 y using age-specific HRs and smoking prevalence and then summing these age-specific estimates to obtain the overall number of deaths due to smoking for that disease. This age-specific method yielded similar results to the one without age-specific estimates, and, thus, the latter method was used, as it provides a tighter 95% CI than the age-specific method.

## Results

A total of 1,223,092 participants were included in the 21 participating cohorts for this study. Because most studies were conducted among adults aged ≥45 y, participants (*n* = 70,812) who did not contribute person-years in the age group ≥45 y were excluded from this analysis. Also excluded (not mutually exclusively) were participants with prior history of cancer or CVD at baseline (*n* = 47,585), with missing data on tobacco smoking (*n* = 38,898) or vital status (*n* = 451), or with less than 1 y of observation after baseline survey (*n* = 30,039). After these exclusions, 1,049,929 participants (510,261 men; 539,668 women) remained (Table 1). Overall, the mean prevalence of tobacco smoking was 65.1% for men and 7.1% for women. Over a mean follow-up of 10.2 y through roughly the mid-2000s for most cohorts, a total of 123,975 deaths were identified in these cohorts.

Compared with never-smokers, a 1.44-fold higher risk (95% CI = 1.37–1.51) of deaths from all causes was observed among male ever-smokers in pooled analyses of all cohorts (Table 2). The estimated HRs related to smoking were slightly higher in Singapore, Republic of Korea, Japan, and Taiwan than in India and mainland China, although 95% CIs overlapped in some of these point estimates (heterogeneity test: *p*<0.001, *I* ^2^ = 89 [95% CI = 85–92]). Among women, ever smoking was associated with a 1.48-fold higher risk (95% CI = 1.38–1.58) of death from any cause. This risk also varied across study populations (heterogeneity test: *p*<0.001, *I* ^2^ = 82 [95% CI = 74–88]). The lowest elevation of risk was observed among Indian women, in which ever smoking was related to a 1.16-fold (95% CI = 0.98–1.36) elevated risk of deaths from all causes. Elevated risk of death was also seen among former smokers, although the risk was lower than among current smokers (Table S1).

**Table 2:** Association of tobacco smoking with risk of death from all causes in selected study populations in Asia.

Population	Men			Women		
	Number of Participants	Number of Deaths	HR (95% CI)^a^	Number of Participants	Number of Deaths	HR (95% CI)^a^
**All cohorts combined**						
Never smoker	177,956	19,353	1.00	501,246	43,067	1.00
Ever smoker	332,305	54,822	1.44 (1.37, 1.51)	38,422	6,733	1.48 (1.38, 1.58)
**India**						
Never smoker	68,866	6,613	1.00	104,223	6,880	1.00
Ever smoker	49,530	6,554	1.31 (1.26, 1.36)	1,378	198	1.16 (0.98, 1.36)
**Mainland China**						
Never smoker	48,664	4,797	1.00	126,085	6,945	1.00
Ever smoker	91,519	9,036	1.30 (1.25, 1.35)	11,154	1,417	1.36 (1.28, 1.45)
**Taiwan**						
Never smoker	5,830	647	1.00	13,696	1,103	1.00
Ever smoker	7,463	1,344	1.44 (1.30, 1.58)	142	17	1.41 (0.85, 2.33)
**Singapore**						
Never smoker	10,875	1,357	1.00	29,645	2,786	1.00
Ever smoker	14,470	3,399	1.58 (1.48, 1.68)	2,724	692	1.75 (1.60, 1.92)
**Republic of Korea**						
Never smoker	4,153	220	1.00	7,567	330	1.00
Ever smoker	14,565	1,180	1.47 (1.26, 1.72)	841	105	1.36 (1.07, 1.73)
**Japan**						
Never smoker	39,108	5,700	1.00	218,448	24,997	1.00
Ever smoker	152,519	33,162	1.49 (1.42, 1.55)	21,892	4,290	1.50 (1.38, 1.63)
**East Asians** b						
Never smoker	108,630	12,721	1.00	395,441	36,161	1.00
Ever smoker	280,536	48,121	1.46 (1.39, 1.54)	36,753	6,521	1.50 (1.40, 1.61)
**South Asians** c						
Never smoker	69,326	6,632	1.00	105,805	6,906	1.00
Ever smoker	51,769	6,701	1.31 (1.26, 1.36)	1,669	212	1.18 (1.01, 1.38)

a Adjusted for age, education, rural/urban resident, marital status, and body mass index; data from participants with <1 y of follow-up are excluded.

Analyses were conducted among those age 45 y or older.

b Including data from mainland China, Taiwan, Singapore, Republic of Korea, and Japan.

c Including data from India and Bangladesh.

Among men, elevated risk of death due to CVD, cancer, and respiratory diseases was statistically significantly associated with ever smoking in virtually all study populations (Table 3). Ever smoking was associated with a 1.35-fold elevated risk (95% CI = 1.26–1.45) of death due to CVD in the analysis that included all cohorts. The risk, however, varied considerably across populations, with the strongest association observed in Taiwan (HR = 1.69; 95% CI = 1.36–2.10) and the weakest association observed in mainland China (HR = 1.17; 95% CI = 1.11–1.25) (heterogeneity test: *p*<0.001, *I* ^2^ = 77 [95% CI = 66–85]). A 1.75-fold elevated risk (95% CI = 1.67–1.85) of death due to cancer in men was associated with ever smoking in the combined analysis of all cohorts. The association with cancer risk was, in general, quite consistent across study populations (heterogeneity test: *p* = 0.76). For death due to respiratory diseases in men, a 1.53-fold elevated risk (95% CI = 1.39–1.69) was associated with ever smoking in the combined analysis of all cohorts, and no statistically significant heterogeneity was identified (*p* = 0.29). Among East Asian women, positive associations were also observed between ever smoking and risk of major cause-specific deaths, with HRs ranging from 1.44 (95% CI = 1.23–1.69) for respiratory diseases to 1.59 for CVD (95% CI = 1.41–1.79) and cancer (95% CI = 1.45–1.75). Heterogeneity tests were statistically significant for cancer (*p*<0.001) and respiratory diseases (*p* = 0.003) but not for CVD (*p* = 0.20). Some of the country-specific risk estimates for East Asian women were not statistically significant because of low smoking prevalence among women in Asia. Among Indian women and all South Asian women combined, the association between ever smoking and risk of cause-specific deaths was weak and statistically nonsignificant.

**Table 3:** Association of tobacco smoking with risk of death from cardiovascular diseases, cancer, or respiratory diseases in selected study populations in Asia.

Population	CVD		Cancer		Respiratory Diseases	
	Number of Deaths^a^	HR (95% CI)^b^	Number of Deaths^a^	HR (95% CI)^b^	Number of Deaths^a^	HR (95% CI)^b^
**Men**						
All cohorts combined	15,381/6,526	1.35 (1.26, 1.45)	17,049/3,818	1.75 (1.67, 1.85)	5,671/1,764	1.53 (1.39, 1.69)
India	2,183/2,275	1.27 (1.18, 1.36)	663/379	1.84 (1.59, 2.13)	825/615	1.50 (1.33, 1.69)
Mainland China	3,378/2,055	1.17 (1.11, 1.25)	3,195/1,227	1.72 (1.60, 1.85)	619/326	1.36 (1.18, 1.57)
Taiwan	291/121	1.69 (1.36, 2.10)	488/208	1.63 (1.38, 1.93)	111/43	1.59 (1.09, 2.32)
Singapore	1,050/476	1.43 (1.28, 1.60)	1,313/451	1.85 (1.66, 2.07)	591/190	1.79 (1.51, 2.13)
Republic of Korea	200/43	1.27 (0.90, 1.80)	543/92	1.66 (1.32, 2.10)	72/11	1.67 (0.82, 3.40)
Japan	8,208/1,544	1.35 (1.27, 1.43)	10,825/1,460	1.77 (1.67, 1.88)	3,437/579	1.55 (1.41, 1.70)
East Asians^c^	13,127/4,239	1.38 (1.28, 1.49)	16,364/3,438	1.75 (1.66, 1.84)	4,830/1,149	1.54 (1.38, 1.72)
South Asians^d^	2,254/2,287	1.26 (1.18, 1.35)	685/380	1.85 (1.59, 2.17)	841/615	1.50 (1.33, 1.69)
**Women**						
All cohorts combined	2,552/13,837	1.54 (1.36, 1.73)	1,752/9,971	1.58 (1.44, 1.74)	655/3,760	1.40 (1.20, 1.63)
India	59/2,316	1.04 (0.77, 1.40)	13/587	1.18 (0.66, 2.10)	29/814	1.05 (0.68, 1.62)
Mainland China	666/2,687	1.48 (1.35, 1.62)	344/1,827	1.56 (1.37, 1.77)	63/323	1.18 (0.89, 1.58)
Taiwan	4/235	1.73 (0.64, 4.71)	12/399	2.94 (1.59, 5.42)	0/63	—e
Singapore	230/969	1.50 (1.28, 1.75)	262/992	2.19 (1.90, 2.54)	112/323	2.13 (1.70, 2.67)
Republic of Korea	35/113	1.24 (0.83, 1.88)	18/79	1.46 (0.83, 2.57)	8/22	1.34 (0.54, 3.31)
Japan	1,511/7,502	1.63 (1.37, 1.95)	1,102/6,083	1.49 (1.39, 1.59)	441/2,212	1.40 (1.26, 1.56)
East Asians^c^	2,486/11,506	1.59 (1.41, 1.79)	1,738/9,380	1.59 (1.45, 1.75)	624/2,943	1.44 (1.23, 1.69)
South Asians^d^	66/2,331	1.07 (0.81, 1.43)	14/591	1.16 (0.66, 2.04)	31/817	1.05 (0.69, 1.61)

a Number of deaths among ever-smokers/never-smokers are presented.

b Adjusted for age, education, rural/urban resident, marital status, and body mass index; data from participants with <1 y of follow-up are excluded.

Analyses were conducted among those age 45 y or older.

c Including data from mainland China, Taiwan, Singapore, Republic of Korea, and Japan.

d Including data from India and Bangladesh.

e HR not estimated because of small sample size.

To quantify risk associated with smoking status and pack-years of smoking, we combined cohorts by ethnic background to improve the stability of point estimates. For men (Table 4) and women (Table 5), risk of total mortality and cause-specific mortality was elevated with increased tobacco smoking among current smokers, measured by pack-years of smoking. Excess deaths were also observed among former smokers, compared with never-smokers, although the risk was lower than for current smokers for deaths due to any cause, CVD, and cancer. A substantially elevated risk of death from respiratory diseases was found among former smokers, particularly in Chinese/Koreans and Indians/Bangladeshis. This excess is probably caused by some smokers quitting smoking after they developed respiratory diseases. Risks associated with smoking status and pack-years of smoking were not estimated for South Asian women because of the small sample size.

**Table 4:** Association of tobacco smoking with risk of death from all causes, cardiovascular diseases, cancer, or respiratory diseases in major Asian male populations.

Population	Tobacco Smoking	Number of Participants	Deaths from All Causes^a^		CVD Deaths^a^		Cancer Deaths^a^		Respiratory Disease Deaths^a^	
			Number of Deaths	HR (95% CI)^b^	Number of Deaths	HR (95% CI)^b^	Number of Deaths	HR (95% CI)^b^	Number of Deaths	HR (95% CI)^b^
**Chinese and Koreans (** ***n*** ** = 155,062)**	**Never**	69,522	7,021	1	2,695	1	1,978	1	570	1
	**Ever**	85,540	9,904	1.48 (1.43, 1.53)	2,921	1.37 (1.29, 1.47)	3,998	1.77 (1.66, 1.88)	1,188	1.75 (1.57, 1.96)
	**Former**	20,256	2,583	1.27 (1.21, 1.34)	866	1.24 (1.14, 1.35)	839	1.27 (1.16, 1.39)	382	1.87 (1.62, 2.15)
	**Current** c	65,258	7,312	1.61 (1.55, 1.68)	2,053	1.47 (1.37, 1.58)	3,156	2.02 (1.89, 2.16)	806	1.71 (1.51, 1.94)
	0–9.9 pack-years	6,928	763	1.34 (1.23, 1.45)	229	1.29 (1.12, 1.49)	285	1.44 (1.26, 1.65)	75	1.41 (1.09, 1.82)
	10–19.9 pack-years	11,307	1,078	1.37 (1.28, 1.47)	309	1.30 (1.14, 1.49)	457	1.65 (1.48, 1.85)	82	1.20 (0.94, 1.55)
	20–29.9 pack-years	13,632	1,461	1.50 (1.41, 1.60)	401	1.37 (1.22, 1.54)	637	1.88 (1.70, 2.08)	167	1.69 (1.40, 2.04)
	≥30 pack-years	33,391	4,010	1.84 (1.75, 1.93)	1,114	1.64 (1.51, 1.79)	1,777	2.42 (2.24, 2.61)	482	2.00 (1.75, 2.30)
**Japanese (** ***n*** ** = 187,002)**	**Never**	39,108	5,700	1	1,544	1	1,460	1	579	1
	**Ever**	147,894	32,214	1.48 (1.43, 1.52)	7,958	1.34 (1.26, 1.42)	10,466	1.76 (1.67, 1.87)	3,343	1.54 (1.41, 1.69)
	**Former**	43,248	8,207	1.24 (1.20, 1.28)	1,939	1.14 (1.06, 1.22)	2,423	1.39 (1.30, 1.49)	975	1.46 (1.31, 1.64)
	**Current** c	104,646	24,007	1.60 (1.55, 1.65)	6,019	1.45 (1.37, 1.54)	8,043	1.94 (1.83, 2.05)	2,368	1.57 (1.43, 1.73)
	0–9.9 pack-years	4,283	708	1.54 (1.42, 1.68)	183	1.58 (1.34, 1.86)	189	1.50 (1.28, 1.76)	63	1.93 (1.47, 2.54)
	10–19.9 pack-years	12,238	2,361	1.65 (1.57, 1.74)	679	1.69 (1.54, 1.86)	701	1.76 (1.60, 1.94)	207	1.55 (1.31, 1.83)
	20–29.9 pack-years	25,045	4,796	1.78 (1.71, 1.86)	1,263	1.77 (1.64, 1.92)	1,547	2.02 (1.88, 2.18)	413	1.73 (1.52, 1.98)
	≥30 pack-years	63,080	16,142	1.58 (1.53, 1.63)	3,894	1.35 (1.27, 1.44)	5,606	2.00 (1.88, 2.12)	1,685	1.55 (1.41, 1.72)
**Indians and Bangladeshis (** ***n*** ** = 101,278)**	**Never**	69,326	6,632	1	2,287	1	380	1	615	1
	**Ever**	31,952	4,500	1.30 (1.23, 1.36)	1,657	1.31 (1.21, 1.43)	520	1.71 (1.44, 2.03)	825	1.46 (1.26, 1.69)
	**Former**	6,860	1,157	1.27 (1.18, 1.37)	399	1.16 (1.03, 1.31)	92	1.78 (1.35, 2.34)	144	1.70 (1.37, 2.10)
	**Current** c	25,092	3,343	1.30 (1.21, 1.39)	1,258	1.41 (1.27, 1.57)	428	1.62 (1.32, 1.99)	681	1.26 (1.05, 1.52)
	0–9.9 pack-years	6,654	597	1.17 (1.06, 1.30)	247	1.32 (1.12, 1.55)	62	1.17 (0.85, 1.62)	71	1.10 (0.81, 1.49)
	10–19.9 pack-years	6,623	704	1.22 (1.11, 1.35)	270	1.35 (1.15, 1.58)	95	1.69 (1.28, 2.24)	101	1.17 (0.89, 1.55)
	20–29.9 pack-years	4,466	610	1.35 (1.22, 1.49)	222	1.39 (1.17, 1.65)	89	1.82 (1.36, 2.46)	88	1.37 (1.05, 1.80)
	≥30 pack-years	7,349	1,432	1.39 (1.28, 1.50)	519	1.51 (1.32, 1.72)	182	1.76 (1.38, 2.25)	222	1.32 (1.06, 1.64)

a Excluding participants with less than 1 y of follow-up.

b Adjusted for age, education, rural/urban resident, marital status, and body mass index.

Analyses were conducted among those age 45 y or older.

c Excluding current smokers with missing information on pack-years of smoking.

**Table 5:** Association of tobacco smoking with risk of death from all causes, cardiovascular diseases, cancer, or respiratory diseases in major East Asian female populations.

Population	Tobacco Smoking	Number of Participants	Deaths from All Causes^a^		CVD Deaths^a^		Cancer Deaths^a^		Respiratory Disease Deaths^a^	
			Number of Deaths	HR (95% CI)^b^	Number of Deaths	HR (95% CI)^b^	Number of Deaths	HR (95% CI)^b^	Number of Deaths	HR (95% CI)^b^
**Chinese and Koreans (** ***n*** ** = 182,640)**	**Never**	176,993	11,164	1	4,004	1	3,297	1	731	1
	**Ever**	5,647	1,028	1.65 (1.53, 1.77)	362	1.57 (1.39, 1.77)	356	1.98 (1.75, 2.24)	128	1.98 (1.61, 2.45)
	**Former**	1,609	305	1.38 (1.22, 1.56)	121	1.35 (1.11, 1.65)	86	1.62 (1.29, 2.04)	28	1.62 (1.08, 2.41)
	**Current** c	4,020	722	1.79 (1.65, 1.95)	241	1.73 (1.50, 2.00)	269	2.15 (1.88, 2.47)	100	2.37 (1.88, 3.00)
	0–9.9 pack-years	1,930	244	1.59 (1.39, 1.82)	79	1.56 (1.24, 1.98)	93	1.86 (1.50, 2.30)	31	2.16 (1.48, 3.16)
	10–19.9 pack-years	757	132	1.77 (1.48, 2.13)	38	1.52 (1.08, 2.14)	55	2.52 (1.90, 3.33)	14	1.91 (1.09, 3.37)
	≥20 pack-years	1,333	346	2.01 (1.79, 2.26)	124	2.01 (1.65, 2.43)	121	2.39 (1.96, 2.91)	55	2.87 (2.13, 3.86)
**Japanese (** ***n*** ** = 239,171)**	**Never**	218,448	24,997	1	7,502	1	6,083	1	2,212	1
	**Ever**	20,723	4,053	1.42 (1.38, 1.48)	1,461	1.46 (1.37, 1.54)	1,043	1.49 (1.39, 1.60)	416	1.39 (1.25, 1.55)
	**Former**	3,957	788	1.24 (1.15, 1.34)	302	1.23 (1.09, 1.39)	203	1.43 (1.24, 1.65)	75	1.13 (0.89, 1.44)
	**Current** c	16,766	3,265	1.48 (1.42, 1.53)	1,159	1.53 (1.43, 1.63)	840	1.51 (1.40, 1.63)	341	1.48 (1.31, 1.66)
	0–9.9 pack-years	6,001	990	1.43 (1.34, 1.52)	339	1.48 (1.32, 1.65)	230	1.35 (1.18, 1.54)	99	1.46 (1.19, 1.79)
	10–19.9 pack-years	4,831	828	1.48 (1.38, 1.59)	296	1.57 (1.39, 1.78)	210	1.46 (1.27, 1.68)	96	1.76 (1.43, 2.18)
	≥20 pack-years	5,934	1,447	1.52 (1.43, 1.60)	524	1.56 (1.43, 1.71)	400	1.70 (1.53, 1.88)	146	1.45 (1.21, 1.72)

a Excluding participants with less than 1 y of follow-up.

b Adjusted for age, education, rural/urban resident, marital status, and body mass index.

Analyses were conducted among those age 45 y or older.

c Excluding current smokers with missing information on pack-years of smoking.

Further analyses were performed to estimate smoking-associated HRs for selected cancers as well as for other common diseases (Table 6). Among men and women, the strongest association with tobacco smoking was lung-cancer mortality: a 3- to 4-fold elevated risk consistently across all populations. In East Asian men, ever smoking was also associated with elevated risk for cancers of the mouth/pharynx/larynx, esophagus, stomach, colorectum, liver, pancreas, and bladder, cancers that have been consistently related to smoking in previous studies. HR estimates for South Asians were statistically nonsignificant or unreliable for several cancers, probably because of small sample sizes. Because of the relatively small sample size of female ever-smokers in South Asia, results are presented for East Asian women only. As in men, risks were elevated for virtually all smoking-related cancers.

**Table 6:** Association of tobacco smoking with risk of cause-specific death by study populations in Asia.

Cause of Death	Men						Women			
	Chinese/Koreans		Japanese		South Asians		Chinese/Koreans		Japanese	
	Number of Deaths^a^	HR^b^ (95% CI)	Number of Deaths^a^	HR^b^ (95% CI)	Number of Deaths^a^	HR^b^ (95% CI)	Number of Deaths^a^	HR^b^ (95% CI)	Number of Deaths^a^	HR^b^ (95% CI)
**Cancer**										
Mouth/pharynx/larynx	290/106	1.95 (1.51, 2.50)	286/36	1.89 (1.28, 2.79)	165/60	1.36 (0.94, 1.98)	16/97	1.99 (1.11, 3.59)	16/81	2.29 (1.28, 4.11)
Esophagus	360/123	1.54 (0.66, 3.57)	625/44	3.05 (2.21, 4.22)	52/9	3.13 (1.43, 6.86)	25/139	0.92 (0.54, 1.57)	21/82	2.62 (1.54, 4.46)
Stomach	855/352	1.43 (1.24, 1.64)	2,440/381	1.48 (1.32, 1.66)	43/16	1.40 (0.67, 2.94)	68/502	1.14 (1.08, 1.52)	191/1,150	1.32 (1.09, 1.59)
Colorectal	490/301	1.13 (0.93, 1.37)	1,123/222	1.22 (1.01, 1.47)	17/11	0.98 (0.40, 2.36)	76/541	1.40 (1.08, 1.83)	124/939	1.11 (0.89, 1.39)
Liver	1,019/460	1.35 (1.19, 1.53)	1,448/198	1.74 (1.48, 2.04)	40/20	1.00 (0.46, 2.16)	67/428	1.75 (1.05, 2.84)	135/621	1.83 (1.50, 2.24)
Pancreas	222/107	1.18 (0.75, 1.86)	658/100	1.60 (1.27, 2.01)	7/6	—c	31/200	1.65 (1.08, 2.53)	98/578	1.59 (1.21, 2.09)
Lung	2,124/374	3.56 (2.45, 5.16)	2,866/164	4.12 (3.49, 4.87)	108/20	3.16 (1.76, 5.69)	291/729	3.34 (2.29, 4.86)	253/714	3.15 (2.70, 3.68)
Bladder	80/30	1.97 (1.26, 3.09)	199/26	1.84 (1.07, 3.16)	12/2	—c	8/36	1.41 (0.56, 3.52)	17/86	1.63 (0.92, 2.90)
Breast							60/507	1.45 (1.05, 1.99)	74/467	1.40 (1.07, 1.84)
Cervix uteri							19/150	1.04 (0.58, 1.88)	28/122	2.09 (1.32, 3.30)
Other	855/432	1.22 (1.07, 1.40)	2,375/457	1.26 (1.12, 1.42)	131/76	1.11 (0.76, 1.62)	118/898	1.41 (1.13, 1.76)	283/1,945	1.17 (1.01, 1.36)
**CVD**										
CHD	1,828/903	1.52 (1.22, 1.90)	2,264/327	1.72 (1.52, 1.95)	1,159/553	1.57 (1.38, 1.78)	343/1,347	1.68 (1.47, 1.92)	391/1,579	1.89 (1.60, 2.23)
Stroke	2,733/1,613	1.19 (1.11, 1.28)	4,193/896	1.19 (1.10, 1.29)	411/261	1.09 (0.90, 1.32)	453/2,390	1.37 (1.16, 1.63)	787/4,075	1.62 (1.27, 2.07)
Other	1,491/877	1.36 (1.09, 1.70)	3,186/589	1.42 (1.26, 1.60)	115/35	1.01 (0.62, 1.64)	379/1,477	1.46 (1.29, 1.67)	636/3,201	1.42 (1.23, 1.64)
**Respiratory disease**										
COPD	820/224	2.05 (1.40, 3.01)	728/75	2.73 (1.93, 3.31)	522/208	1.28 (1.05, 1.57)	95/223	2.82 (1.18, 6.72)	95/262	2.10 (1.18, 3.72)
Other	755/457	1.09 (0.93, 1.29)	3,158/606	1.42 (1.29, 1.55)	77/45	1.07 (0.68, 1.68)	131/643	1.46 (1.13, 1.88)	405/2,246	1.38 (1.19, 1.60)
**Tuberculosis**	151/75	0.88 (0.64, 1.23)	56/14	0.66 (0.27, 1.60)	225/65	1.81 (1.21, 2.70)	12/76	0.90 (0.43, 1.85)	9/21	2.43 (0.92, 6.46)
**All other known causes**	3,369/1,956	1.11 (1.02, 1.20)	5,935/1,206	1.23 (1.13, 1.34)	1,342/818	1.09 (0.97, 1.21)	542/3,619	1.14 (1.00, 1.29)	943/5,307	1.35 (1.12, 1.62)

a Number of deaths among ever-smokers/never-smokers are presented.

b HRs estimated for ever-smokers compared with never-smokers and adjusted for age, education, rural/urban residence, marital status, and body mass index; data from participants with <1 y of follow-up are excluded.

Analyses were conducted among those age 45 y or older.

c HR not estimated because of small sample size.

CHD, coronary heart disease; COPD, chronic obstructive pulmonary disease.

Among East Asian men and women, risks of death associated with smoking were elevated for coronary heart disease, stroke, and chronic obstructive pulmonary disease. Among South Asian men, the association was statistically significant for coronary heart disease and chronic obstructive pulmonary disease but not for stroke. Smoking was associated with elevated risk of death due to tuberculosis in South Asians. However, no association between smoking and tuberculosis was found in East Asians.

Among men ≥45 y, approximately 15.8% (95% CI = 14.3%–17.2%) of deaths (1.34 million [95% CI = 1.21–1.46 million]) from all causes in 2004 were attributable to tobacco smoking in these seven countries/regions combined (Table 7). Smoking-associated PARs for all-cause mortality were higher in Japan (27.7%), Republic of Korea (26.9%), and Singapore (24.8%) than in mainland China (16.2%), India (11.5%), Taiwan (19.7%), and Bangladesh (14.3%). Among women ≥45 y, tobacco smoking accounted for ∼3.3% (95% CI = 2.6%–4.0%) of total deaths (239,000 [95% CI = 188,300–289,700]) in the seven countries/regions combined in 2004.

**Table 7:** Smoking prevalence, population attributable risk, and number of deaths due to tobacco smoking in selected Asian populations.

Population	Men			Women		
	Smoking Prevalence (Percent)	PAR (Percent)	Number of Deaths (in Thousands)	Smoking Prevalence (Percent)	PAR (Percent)	Number of Deaths (in Thousands)
Bangladesh	53.8^a^	14.3 (12.2, 16.4)^b^	46.4 (39.6, 53.3)	3.0^a^	0.5 (0.0, 1.1)^b^	1.5 (0, 3.4)
India	41.8	11.5 (9.8, 13.2)	378.8 (322.8, 434.8)	1.3	0.2 (0.0, 0.4)	6.9 (0, 11.0)
Mainland China	65.3	16.2 (14.0, 18.5)	675.7 (583.9, 771.6)	8.1	2.9 (2.2, 3.5	104.6 (79.4, 126.3)
Taiwan^c^	56.1	19.7 (14.6, 24.7)	18.4 (13.6, 23.0)	4.2^a^	1.7 (0.0, 4.6)	1.0 (0, 2.7)
Singapore	57.1	24.8 (21.4, 28.1)	2.5 (2.1, 2.8)	8.4	6.0 (4.8, 7.1)	0.5 (0.4, 0.6)
Republic of Korea	77.8	26.9 (17.6, 36.3)	37.8 (24.8, 51.1)	6.1^a^	2.1 (0.2, 4.0)	2.4 (0.2, 4.5)
Japan	79.6	27.7 (25.9, 29.5)	143.7 (134.3, 153.0)	9.1	3.7 (3.3, 4.2)	16.8 (15.0, 19.1)
East Asians^d^	67.3	18.0 (15.9, 20.1)	869.4 (768.0, 970.9)	8.1	2.9 (2.0, 3.9)	122.8 (84.7, 165.1)
South Asians^d^	42.8	11.7 (10.0, 13.4)	424.6 (362.9, 486.3)	1.6	0.3 (0.0, 0.6)	9.2 (0, 18.4)
All populations^d^	58.6	15.8 (14.3, 17.2)	1,336.5 (1,209.7, 1,455.0)	5.8	3.3 (2.6, 4.0)	239.0 (188.3, 289.7)

Estimates are provided for populations age 45 y or older.

a Because of the small sample size in the current study for these populations, data for smoking prevalence rates were obtained from other sources: Bangladeshi men and women: [12], Taiwanese women: [19], and Korean women: [34].

b PARs were estimated using HRs derived from all South Asian cohorts combined because of unstable HR estimates using Bangladeshi data alone.

c Mortality data for Taiwan were obtained from http://www.mohw.gov.tw/CHT/Ministry/Index.aspx.

d PARs were estimated using weighted HRs and smoking prevalence of the study populations.

Thus, the number of deaths attributable to smoking in these populations may not be equal to the sum of the numbers of deaths from the countries in the population areas. East Asia: mainland China, Taiwan, Singapore, Republic of Korea, and Japan. South Asia: Bangladesh and India. All populations: all seven countries/regions listed above.

Among men aged ≥45 y, ∼11.4% (95% CI = 9.1%–13.8%), 30.5% (95% CI = 27.4%–33.6%), and 19.8% (95% CI = 14.8%–24.8%) of deaths due to CVD, cancer, and respiratory diseases, respectively, were attributable to tobacco smoking in 2004 (Table 8). Smoking-associated PARs for cause-specific mortality were also higher, in general, in Japan, Republic of Korea, and Singapore than in the other study populations. Overall, 60.5% (95% CI = 54.5%–66.4%) of lung cancer deaths in men were attributable to tobacco smoking. Data for women are presented for mainland China, Japan, and all East Asians combined, because of the small sample size for other groups. Smoking-associated PARs were much smaller in women than in men: 1.7% (95% CI = 0%–4.0%), 3.7% (95% CI = 2.4%–5.0%), and 4.6% (95% CI = 3.3%–5.8%) for deaths due to respiratory diseases, CVD, and cancer, respectively. Nevertheless, ∼16.7% (95% CI = 13.3%–20.0%) of lung-cancer deaths in East Asian women ≥45 y were attributable to tobacco smoking in 2004.

**Table 8:** Population-attributable risk and number of cause-specific deaths due to tobacco smoking in selected Asian populations.

Population	CVD		All Cancers		Lung Cancer		Respiratory Disease	
	PAR (Percent)	Number of Deaths (in Thousands)	PAR (Percent)	Number of Deaths (in Thousands)	PAR (Percent)	Number of Deaths (in Thousands)	PAR (Percent)	Number of Deaths (in Thousands)
**Men**								
Bangladesh	12.3^a^	16.4 (11.6, 21.3)	31.3^a^	10.8 (8.4, 13.1)	61.1^a^	8.6 (6.6, 10.5)	21.2^a^	6.4 (4.6, 8.3)
India	10.0	130.8 (91.5, 170.0)	26.1	85.6 (65.2, 105.6)	55.0	30.1 (22.1, 38.0)	17.3	69.2 (48.0, 90.0)
Mainland China	10.2	159.4 (101.5, 215.5)	32.0	325.1 (287.6, 361.7)	62.5	154.5 (143.6, 165.7)	19.0	141.0 (78.7, 202.6)
Taiwan	27.9	1.3 (0.8, 1.7)	26.2	7.1 (4.8, 9.4)	58.9	3.4 (3.1, 3.6)	24.9	1.1 (0.3, 1.9)
Singapore	19.8	0.7 (0.5, 0.9)	32.8	0.9 (0.8, 1.1)	59.3	0.5 (0.4, 0.5)	31.2	0.2 (0.1, 0.2)
Republic of Korea	17.3	6.1 (0, 14.3)	34.0	17.0 (10.5, 23.6)	66.5	8.4 (7.8, 8.9)	34.4	3.1 (0, 6.7)
Japan	21.7	31.4 (26.1, 36.9)	38.0	72.0 (66.3, 77.9)	67.0	29.0 (27.2, 30.8)	30.4	9.2 (7.5, 10.9)
East Asians^b^	12.2	212.4 (156.7, 268.2)	32.7	410.6 (369.1, 453.2)	63.2	191.6 (177.6, 205.8)	21.0	164.1 (108.6, 220.4)
South Asians^b^	10.0	144.5 (101.2, 189.3)	26.6	97.1 (74.5, 119.7)	55.5	38.6 (28.5, 48.7)	17.6	75.8 (52.6, 98.7)
All populations^b^	11.4	363.3 (290.0, 439.7)	30.5	494.3 (444.0, 544.5)	60.5	225.5 (203.1, 247.5)	19.8	240.1 (179.5, 300.7)
**Women**								
Mainland China	3.7	58.5 (42.7, 75.9)	4.3	25.5 (17.2, 34.4)	13.9	15.9 (12.0, 19.7)	1.5	11.7 (0, 32.8)
Japan	4.0	6.2 (5.5, 7.0)	4.3	5.4 (4.3, 6.5)	15.4	2.5 (1.9, 3.1)	3.5	0.7 (0.5, 1.0)
East Asians^b^	3.7	66.3 (43.0, 89.6)	4.6	35.1 (25.2, 44.2)	16.7	23.0 (18.3, 27.5)	1.7	13.8 (0, 32.4)

Estimates are provided for populations age 45 y or older.

a PARs were estimated using HRs derived from all South Asian cohorts combined because of unstable HR estimates using Bangladeshi data alone.

b PARs were estimated using weighted HRs and smoking prevalence of the study populations.

Thus, the number of deaths attributable to smoking in these populations may not be equal to the sum of the numbers of deaths from countries in the population areas. East Asia: mainland China, Taiwan, Singapore, Republic of Korea, and Japan. South Asia: Bangladesh and India. All populations: all seven countries/regions listed above.

## Discussion

Similar to studies conducted in Europe and North America [1]–[5],[9],[10], we found that tobacco smoking is associated with substantially elevated risk of total and cause-specific mortality. This study analyzed individual-level data from seven Asian countries/regions, using a uniform analytic approach, which enabled comparisons of smoking-associated HRs and PARs across these countries/regions. This study provides strong evidence that tobacco smoking is a major cause of death in Asia and underscores the importance and urgency of implementing comprehensive tobacco control programs for disease prevention in this populous continent.

Most smoking-associated RR estimates in this study were 1.3–1.5 for all-cause mortality, comparable to most estimates from previous studies conducted in Asia [14]–[21]. These RRs, however, are substantially lower than those from studies conducted in Europe and North America, where >2-fold elevated risk for all-cause mortality is typically reported for current smokers [1]–[3],[5],[9],[10]. Among specific causes of mortality evaluated in this study, lung cancer showed the strongest association with tobacco smoking, with estimated HRs of 3.0 to 4.0, approximately one-third of the risk observed in most studies conducted in Western countries [1]–[3],[5],[9],[10]. The smaller effect of smoking on mortality in Asia compared with Western countries could be partly explained by the fact that widespread tobacco smoking in most Asian countries began several decades later than in Europe and North America, and thus many Asian countries are still in the early stages of a tobacco epidemic; many smokers in the population started smoking tobacco at a late age and smoke a small number of cigarettes daily [3],[12]. In the British Doctors Study, a 1.6-fold elevated risk of all-cause mortality was observed among smokers in early years of follow-up (1951–1971) [26], close to the effect size estimated in this study. In later follow-up (1971–1991), the RR rose to 2.1. A recent Japanese study showed a clear birth-cohort effect: male smokers born before 1890 started smoking at a later age and smoked fewer cigarettes daily than those born in 1940–1945 [27]. As a result, the association of smoking with risk of all-cause mortality was weaker in the older cohort (RR = 1.24) than the younger cohort (RR = 1.92). Our study showed a clear dose–response relationship between pack-years of smoking and risk of all-cause and cause-specific mortality. It is likely that, with maturation of the tobacco epidemic in Asia and lack of effective tobacco control, more smokers will accumulate much higher pack-years of smoking, and, thus, smoking-associated RRs will rise, mirroring the trend in the US and Europe.

Several previous studies have estimated the burden of disease due to tobacco smoking in a specific Asian country/region [14],[16],[17],[19],[20] (Table S2). However, most previous estimates for smoking-associated RRs and PARs were derived from either a single cohort study [14],[19] or a retrospective case–control study [16]–[18]. Not all previous studies had detailed demographic and risk-factor information to adequately adjust for potential confounders when estimating risks. Three previous studies conducted in mainland China and Taiwan provided somewhat lower estimates of total male deaths due to smoking than our estimates, perhaps because these studies were conducted during even earlier stages of the tobacco epidemic in these populations, resulting in smaller PARs [14],[16],[19]. For India, however, the estimate of male deaths attributable to tobacco smoking from a previous study (20% of total male deaths) [17] was substantially higher than the estimate from our study (11.5% of total male deaths). To our knowledge, no study has been previously conducted in Bangladesh, the Republic of Korea, or Singapore; thus, our study provides, for the first time, direct estimates of deaths due to tobacco smoking in these countries. Despite methodological differences between this and previous studies, all studies conducted to date have shown that an alarming proportion of deaths are caused by tobacco smoking.

In this study, some estimates among women are unstable because of very low smoking prevalence. Although not all participating cohorts are representative of the general population, smoking-associated RRs estimated in this study, are, in general, comparable to those from previous studies. Furthermore, smoking-associated RRs estimated in multiple cohorts within the same country are, in general, comparable. It is difficult to find national survey data consistent with the definitions, time period, and age groups of our study for all seven countries/regions in our analysis. Many national surveys used a smaller sample than our study, providing unstable smoking prevalence estimates. Therefore, we chose to use smoking prevalence estimates from our own study to estimate PARs: smoking-associated RRs were estimated based on exposure history of the same group of individuals, which should provide better estimates of disease burden due to tobacco smoking in the study population than using data from external sources. Smoking prevalence has declined recently in several high-income Asian countries. However, given the long latency of chronic diseases, typically 15 y and longer, it is reasonable to use smoking prevalence rates assessed in the 1990s to estimate number of deaths due to tobacco smoking in 2004. As most of the cohort studies included in this study were conducted among adults aged ≥45 y, we were unable to estimate the impact of active tobacco smoking in people younger than 45 y old. Again, because of the long latency of chronic diseases, most of the smoking-related diseases tend to occur later in life.

We estimated smoking-associated PARs and numbers of deaths due to tobacco smoking in 2004. As many Asian countries, such as China and India, are still in the early stage of tobacco epidemics, the number of deaths due to tobacco smoking in more recent years in these countries is likely to be larger than that estimated in this study.

Data on secondhand tobacco-smoke exposure was not available in this study. Secondhand smoke has been linked to an elevated risk of multiple chronic diseases [2],[28],[29]. It has been estimated that approximately 603,000 deaths worldwide may be due to secondhand smoke [29]. We also were unable to evaluate smokeless tobacco, a risk factor for oral cancer and several other chronic diseases [30],[31]. Smokeless tobacco use is common in India and Bangladesh, especially among women in these countries. Some individuals who had secondhand tobacco-smoke exposure or used smokeless tobacco may be included in the reference group, which may result in an underestimate of the risk associated with active tobacco smoking. Furthermore, in our study, RRs associated with tobacco smoking were estimated primarily based on the time period from the early 1990s to the mid-2000s. Because smoking-associated risk of death is likely to increase with the maturation of the tobacco epidemic, the total number of deaths due to smoking in 2004 may be underestimated using this set of RRs. Therefore, the true impact of tobacco smoking on mortality in these Asian countries is likely to be even larger than estimated here. Despite some limitations mentioned above, our study provides perhaps the best estimates of tobacco-associated deaths to date in these Asian countries/regions.

Over the past 50 years, the landscape of tobacco smoking has changed dramatically around the world. Smoking prevalence has declined sharply in many high-income countries, resulting in a recent decrease in smoking-related deaths, particularly among men [3],[8]. Conversely, prevalence of tobacco use remains high in China, India, and other low- and middle-income countries. As the tobacco epidemic grows in these countries, we anticipate that an increasing number of deaths will be attributable to tobacco smoking in Asia in the coming years. Even in more well-developed Asian countries such as Japan and Republic of Korea, where smoking rates have recently declined, the full impact of tobacco smoking on mortality is unlikely to be seen soon because, as noted above, smokers in recent birth cohorts tend to smoke more and start smoking earlier, elevating their risk of smoking-associated deaths, and because of the long latency of the diseases associated with smoking, these deaths will not accrue immediately. Our study shows that tobacco smoking is a major cause of death in Asia, accounting for ∼1.6 million deaths of adults ≥45 y in 2004 in the seven countries/regions in this analysis. If the remaining 29% of the Asian population is experiencing a tobacco epidemic similar to that of these seven countries/regions, we estimate that, in 2004, >2 million deaths in Asia were attributable to tobacco smoking. Thus, of the 5 million deaths currently attributable to active tobacco smoking worldwide [32], nearly 45% occur in Asia. Our study provides sobering evidence that stresses the urgency of implementing comprehensive tobacco control programs in Asia, as recommended by the WHO Framework Convention on Tobacco Control [33]. Tobacco control should be among the top priorities in Asia to reduce the burden of disease.

## Supporting Information

Table S1 **Association of tobacco smoking status (former or current) with risk of death from all causes in selected study populations in Asia.**
(DOC)

Table S2 **Population-attributable risk and number of deaths due to smoking in major Asian populations estimated in previous studies.**
(DOC)

Text S1 **Descriptions of participating cohorts.**
(DOC)

## Abbreviations

CVD: cardiovascular disease
HR: hazard ratio
PAR: population attributable risk
RR: relative risk
