# Supplementary material for: Burden of Total and Cause-Specific Mortality Related to Tobacco Smoking among Adults Aged ≥45 Years in Asia: A Pooled Analysis of 21 Cohorts
Source: PLoS Med. 2014 Apr 22;11(4):e1001631. doi: 10.1371/journal.pmed.1001631 (PMC3995657; doi:10.1371/journal.pmed.1001631)
Supplement: Table S1 — Association of tobacco smoking status (former or current) with risk of death from all causes in selected study populations in Asia. (DOC) [file pmed.1001631.s001.doc]

**Supporting Table S1**

**Association of Tobacco Smoking with Total and Cause-Specific Mortality in Adults over 45 Years Old in Asia: A Pooled Analysis of 21 Cohorts**

Wei Zheng, Dale F. McLerran, Betsy A. Rolland, Zhenming Fu, Paolo Boffetta, Jiang He, Prakash Chandra Gupta, Kunnambath Ramadas, Shoichiro Tsugane, Fujiko Irie, Akiko Tamakoshi, Yu-Tang Gao, Woon-Puay Koh, Xiao-Ou Shu, Kotaro Ozasa, Yoshikazu Nishino, Ichiro Tsuji, Hideo Tanaka, Chien-Jen Chen, Jian-Min Yuan, Yoon-Ok Ahn, Keun-Young Yoo, Habibul Ahsan, Wen-Harn Pan, You-Lin Qiao; Dongfeng Gu, Mangesh Suryakant Pednekar, Catherine Sauvaget, Norie Sawada, Toshimi Sairenchi, Gong Yang, Renwei Wang, Yong-Bing Xiang, Waka Ohishi, Masako Kakizaki, Takashi Watanabe, Isao Oze, San-Lin You, Yumi Sugawara, Lesley M. Butler, Dong-Hyun Kim, Sue K. Park, Faruque Parvez, Shao-Yuan Chuang; Jin-Hu Fan; Chen-Yang Shen, Yu Chen, Eric J. Grant, Jung Eun Lee, Rashmi Sinha, Keitaro Matsuo, Mark Thornquist, Manami Inoue, Ziding Feng, Daehee Kang, John D. Potter

Corresponding author’s contact information:

Wei Zheng, M.D., Ph.D.

Vanderbilt Epidemiology Center

Vanderbilt University Medical Center

2525 West End Avenue, 8^th^ Floor

Nashville, TN 37203-1738

E-mail: [wei.zheng@vanderbilt.edu](mailto:wei.zheng@vanderbilt.edu)

| **Supplementary Table S1** **Association of tobacco smoking status (former or current) with risk of death from all causes in selected study populations in Asia** | | | | | | | |
| --- | --- | --- | --- | --- | --- | --- | --- |
|  | **Men** | | |  | **Women** | | |
| **Populations** | **No. of subjects** | **No. of deaths** | **HR  (95% CI)** ^a^ |  | **No. of subjects** | **No. of deaths** | **HR  (95% CI)** ^a^ |
| All cohorts combined |  |  |  |  |  |  |  |
| Never | 177,956 | 19,353 | 1.00 |  | 501,246 | 43,067 | 1.00 |
| Former  Current | 70,364  261,915 | 11,947  42,866 | 1.26 (1.21, 1.31)  1.52 (1.42,1.63) |  | 5,798  32,606 | 1,115  5,617 | 1.30 (1.20, 1.40)  1.52 (1.41, 1.65) |
| India |  |  |  |  |  |  |  |
| Never | 68,866 | 6,613 | 1.00 |  | 104,223 | 6,880 | 1.00 |
| Former  Current | 6,399  43,131 | 1,116  5,438 | 1.29 (1.20, 1.39)  1.31 (1.26, 1.37) |  | 112  1,266 | 15  183 | 1.17 (0.63, 2.18)  1.16 (0.99, 1.37) |
| Mainland China |  |  |  |  |  |  |  |
| Never | 48,664 | 4,797 | 1.00 |  | 126,085 | 6,945 | 1.00 |
| Former  Current | 9,416  82,103 | 1,074  7,962 | 1.37 (1.28, 1.48)  1.29 (1.24, 1.34) |  | 707  10,447 | 115  1302 | 1.36 (1.11, 1.66)  1.36 (1.28, 1.45) |
| Taiwan |  |  |  |  |  |  |  |
| Never | 5,830 | 647 | 1.00 |  | 13,696 | 1,103 | 1.00 |
| Former  Current | 872  6,565 | 158  1,177 | 1.12 (0.93, 1.34)  1.49 (1.35, 1.65) |  | 9  115 | 2  14 | 2.20 (0.30, 16.1)  1.54 (0.90, 2.63) |
| Singapore |  |  |  |  |  |  |  |
| Never | 10,875 | 1,357 | 1.00 |  | 29,645 | 2,786 | 1.00 |
| Former  Current | 5,143  9,327 | 1,042  2,357 | 1.19 (1.10, 1.30)  1.85 (1.72, 1.98) |  | 718  2,006 | 171  521 | 1.42 (1.20, 1.67)  1.89 (1.71, 2.08) |
| Republic of Korea |  |  |  |  |  |  |  |
| Never | 4,153 | 220 | 1.00 |  | 7,567 | 330 | 1.00 |
| Former  Current | 4,825  9,740 | 309  871 | 1.23 (1.03, 1.48)  1.60 (1.37, 1.88) |  | 175  666 | 17  88 | 1.20 (0.72, 2.00)  1.39 (1.08, 1.80) |
| Japan |  |  |  |  |  |  |  |
| Never | 39,108 | 5,700 | 1.00 |  | 218,448 | 24,997 | 1.00 |
| Former  Current | 43,248  109,271 | 8,207  24,955 | 1.24 (1.18, 1.30)  1.63 (1.54, 1.72) |  | 3,957  17,935 | 788  3,502 | 1.26 (1.15, 1.37)  1.56 (1.43, 1.70) |
| East Asians^b^ |  |  |  |  |  |  |  |
| Never | 108,630 | 12,721 | 1.00 |  | 395,441 | 36,161 | 1.00 |
| Former  Current | 63,504  217,006 | 10,790  37,322 | 1.26 (1.20, 1.31)  1.56 (1.46, 1.67) |  | 5,566  31,169 | 1,093  5,427 | 1.29 (1.20, 1.39)  1.56 (1.45, 1.68) |
| South Asians^c^ |  |  |  |  |  |  |  |
| Never | 69,326 | 6,632 | 1.00 |  | 105,805 | 6,906 | 1.00 |
| Former  Current | 6,860  44,909 | 1,157  5,544 | 1.29 (1.20, 1.39)  1.31 (1.25, 1.37) |  | 232  1,437 | 22  190 | 1.42 (0.85, 2.39)  1.18 (1.00, 1.38) |
| ^a^ Adjusted for age, education, rural/urban resident, marital status, and body mass index; data from subjects with less than one year (<1 year) of follow-up are excluded. Analyses were conducted among those age 45 years or older.  ^b^ Including data from Mainland China, Taiwan, Singapore, Republic of Korea and Japan  ^c^ Including data from India and Bangladesh | | | | | | | |
