# Supplementary material for: Burden of Total and Cause-Specific Mortality Related to Tobacco Smoking among Adults Aged ≥45 Years in Asia: A Pooled Analysis of 21 Cohorts
Source: PLoS Med. 2014 Apr 22;11(4):e1001631. doi: 10.1371/journal.pmed.1001631 (PMC3995657; doi:10.1371/journal.pmed.1001631)
Supplement: Table S2 — Population-attributable risk and number of deaths due to smoking in major Asian populations estimated in previous studies. (DOC) [file pmed.1001631.s002.doc]

**Supporting Table S2**

**Association of Tobacco Smoking with Total and Cause-Specific Mortality in Adults over 45 Years Old in Asia: A Pooled Analysis of 21 Cohorts**

Wei Zheng, Dale F. McLerran, Betsy A. Rolland, Zhenming Fu, Paolo Boffetta, Jiang He, Prakash Chandra Gupta, Kunnambath Ramadas, Shoichiro Tsugane, Fujiko Irie, Akiko Tamakoshi, Yu-Tang Gao, Woon-Puay Koh, Xiao-Ou Shu, Kotaro Ozasa, Yoshikazu Nishino, Ichiro Tsuji, Hideo Tanaka, Chien-Jen Chen, Jian-Min Yuan, Yoon-Ok Ahn, Keun-Young Yoo, Habibul Ahsan, Wen-Harn Pan, You-Lin Qiao; Dongfeng Gu, Mangesh Suryakant Pednekar, Catherine Sauvaget, Norie Sawada, Toshimi Sairenchi, Gong Yang, Renwei Wang, Yong-Bing Xiang, Waka Ohishi, Masako Kakizaki, Takashi Watanabe, Isao Oze, San-Lin You, Yumi Sugawara, Lesley M. Butler, Dong-Hyun Kim, Sue K. Park, Faruque Parvez, Shao-Yuan Chuang; Jin-Hu Fan; Chen-Yang Shen, Yu Chen, Eric J. Grant, Jung Eun Lee, Rashmi Sinha, Keitaro Matsuo, Mark Thornquist, Manami Inoue, Ziding Feng, Daehee Kang, John D. Potter

Corresponding author’s contact information:

Wei Zheng, M.D., Ph.D.

Vanderbilt Epidemiology Center

Vanderbilt University Medical Center

2525 West End Avenue, 8^th^ Floor

Nashville, TN 37203-1738

E-mail: [wei.zheng@vanderbilt.edu](mailto:wei.zheng@vanderbilt.edu)

| **Supplementary Table S2. Population-attributable risk and number of deaths due to smoking in major Asian populations estimated in previous studies** | | | | | | | | | | | | | | | | | | | | | | | | | |
| --- | --- | --- | --- | --- | --- | --- | --- | --- | --- | --- | --- | --- | --- | --- | --- | --- | --- | --- | --- | --- | --- | --- | --- | --- | --- |
|  | **Men** | | | | | | | | | | |  | | **Women** | | | | | | | | | | | **Study**  **Populations**  **(age group, year)** |
|  | **All causes** | |  | **CVD** | |  | **All cancer** | |  | **Lung cancer** | |  | **All causes** | | |  | **CVD** | |  | **All cancer** | |  | **Lung cancer** | |
|  | **PAR (%)** | **No.*** |  | **PAR (%)** | **No.*** |  | **PAR (%)** | **No.*** |  | **PAR (%)** | **No.*** |  | **PAR (%)** | | **No.*** |  | **PAR (%)** | **No.*** |  | **PAR (%)** | **No.*** |  | **PAR (%)** | **No.*** |
| **Mainland China** |  |  |  |  |  |  |  |  |  |  |  |  |  | |  |  |  |  |  |  |  |  |  |  |  |
| **Current study** | 16.2 | 675.7 |  | 10.2 | 159.4 |  | 32.0 | 325.1 |  | 62.5 | 154.5 |  | 2.9 | | 104.6 |  | 3.7 | 58.5 |  | 4.3 | 25.5 |  | 13.9 | 15.9 | 45+ years in 2004 |
| **Liu et al (1998)^1^** | 12.6 | 500.0 |  | NA | NA |  | 24.4 | 193.0 |  | 52.3 | 75.0 |  | 3.2 | | 102.0 |  | 1.0^a^ | 10.0^a^ |  | 4.1 | 21.0 |  | 20.0 | 13.0 | 30+ years in 1990 |
| **Gu et al (2009)^2^** | 12.9 | 538.0 |  | 10.7 | 126.6 |  | 28.0 | 240.4 |  | 50.6 | 113.0 |  | 3.1 | | 134.8 |  | 2.1 | 19.6 |  | 5.7 | 27.8 |  | 14.8 | 16.0 | 40+ years in 2005 |
| **India** |  |  |  |  |  |  |  |  |  |  |  |  |  | |  |  |  |  |  |  |  |  |  |  |  |
| **Current study** | 11.5 | 378.8 |  | 10.0 | 130.8 |  | 26.1 | 85.6 |  | 55.0 | 30.1 |  | 0.2 | | 6.9 |  | NA | NA |  | NA | NA |  | NA | NA | 45+ years in 2004 |
| **Jha et al (2008)^3^** | 20.0 | 579.0 |  | 20.0 | 110.0 |  | 32.0 | 67.0 |  | NA | NA |  | 5.0 | | 93.0 |  | 3.0 | 8.0 |  | 4.0 | 9.0 |  | NA | NA | 30-69 years in 2010 |
| **Bangladesh** |  |  |  |  |  |  |  |  |  |  |  |  |  | |  |  |  |  |  |  |  |  |  |  |  |
| **Current study** | 14.3 | 46.4 |  | 12.3 | 16.4 |  | 31.3 | 10.8 |  | 61.1 | 8.6 |  | 0.5 | | 1.5 |  | NA | NA |  | NA | NA |  | NA | NA | 45+ years in 2004 |
| **Japan** |  |  |  |  |  |  |  |  |  |  |  |  |  | |  |  |  |  |  |  |  |  |  |  |  |
| **Current study** | 27.7 | 143.7 |  | 21.7 | 31.4 |  | 38.0 | 72.0 |  | 67.0 | 29.0 |  | 3.7 | | 16.8 |  | 4.0 | 6.7 |  | 4.3 | 5.4 |  | 15.4 | 2.5 | 45+ years in 2004 |
| **Katanoda(2008)^4^** | 27.8 | 163.0 |  | 23.0 | 25.8^a^ |  | 38.6 | 68.3^a^ |  | 69.2 | 32.5^a^ |  | 6.7 | | 33.0 |  | 8.0 | 9.7^a^ |  | 5.2 | 5.9^a^ |  | 19.8 | 3.4^a^ | 40+ years in 2005 |
| **Republic of Korea** |  |  |  |  |  |  |  |  |  |  |  |  |  | |  |  |  |  |  |  |  |  |  |  |  |
| **Current study** | 26.9 | 37.8 |  | 17.3 | 6.1 |  | 34.0 | 17.0 |  | 66.5 | 8.4 |  | 2.1 | | 2.4 |  | NA | NA |  | NA | NA |  | NA | NA | 45+ years in 2004 |
| **Taiwan** |  |  |  |  |  |  |  |  |  |  |  |  |  | |  |  |  |  |  |  |  |  |  |  |  |
| **Current study** | 19.7 | 18.4 |  | 27.9 | 1.3 |  | 26.2 | 7.1 |  | 58.9 | 3.4 |  | 1.7 | | 1.0 |  | NA | NA |  | NA | NA |  | NA | NA | 45+ years in 2004 |
| **Liaw et al (1998)^5^** | 13.9 | 58.5 |  | 30.2 | 3.2 |  | 21.3 | NA |  | 59.3 | 3.1 |  | 3.3 | | 37.4 |  | NA | NA |  | 2.9 | NA |  | 9.8 | 1.2 | 40+ years in 1994 |
| **Singapore** |  |  |  |  |  |  |  |  |  |  |  |  |  | |  |  |  |  |  |  |  |  |  |  |  |
| **Current study** | 24.8 | 2.5 |  | 19.8 | 0.7 |  | 32.8 | 0.9 |  | 59.3 | 0.5 |  | 6.0 | | 0.5 |  | NA | NA |  | NA | NA |  | NA | NA | 45+ years in 2004 |
| *No. of deaths (in thousands) | | | | | | | | | | | | | | | | | | | | | | | | | |
| 1. Liu BQ, Peto R, Chen ZM, Boreham J, Wu YP, Li JY, et al. Emerging tobacco hazards in China: 1. Retrospective proportional mortality study of one million deaths. *BMJ* 1998;317(7170):1411-22. 2. Gu D, Kelly TN, Wu X, Chen J, Samet JM, Huang J-f, et al. Mortality Attributable to Smoking in China. *N Engl J Med* 2009;360(2):150-59. 3. Jha P, Jacob B, Gajalakshmi V, Gupta PC, Dhingra N, Kumar R, et al. A Nationally Representative Case/Control Study of Smoking and Death in India. *N Engl J Med* 2008;358(11):1137-47. 4. Katanoda K, Marugame T, Saika K, Satoh H, Tajima K, Suzuki T, et al. Population attributable fraction of mortality associated with tobacco smoking in Japan: a pooled analysis of three large-scale cohort studies. *J Epidemiol* 2008;18(6):251-64. 5. Liaw KM, Chen CJ. Mortality attributable to cigarette smoking in Taiwan: a 12-year follow-up study. *Tob Control.* 1998;7(2):141-8. | | | | | | | | | | | | | | | | | | | | | | | | | |
