# Supplementary material for: Burden of Total and Cause-Specific Mortality Related to Tobacco Smoking among Adults Aged ≥45 Years in Asia: A Pooled Analysis of 21 Cohorts
Source: PLoS Med. 2014 Apr 22;11(4):e1001631. doi: 10.1371/journal.pmed.1001631 (PMC3995657; doi:10.1371/journal.pmed.1001631)
Supplement: Text S1 — Descriptions of participating cohorts. (DOC) [file pmed.1001631.s003.doc]

**Text S1**

**Association of Tobacco Smoking with Total and Cause-Specific Mortality in Adults over 45 Years Old in Asia: A Pooled Analysis of 21 Cohorts**

Wei Zheng, Dale F. McLerran, Betsy A. Rolland, Zhenming Fu, Paolo Boffetta, Jiang He, Prakash Chandra Gupta, Kunnambath Ramadas, Shoichiro Tsugane, Fujiko Irie, Akiko Tamakoshi, Yu-Tang Gao, Woon-Puay Koh, Xiao-Ou Shu, Kotaro Ozasa, Yoshikazu Nishino, Ichiro Tsuji, Hideo Tanaka, Chien-Jen Chen, Jian-Min Yuan, Yoon-Ok Ahn, Keun-Young Yoo, Habibul Ahsan, Wen-Harn Pan, You-Lin Qiao; Dongfeng Gu, Mangesh Suryakant Pednekar, Catherine Sauvaget, Norie Sawada, Toshimi Sairenchi, Gong Yang, Renwei Wang, Yong-Bing Xiang, Waka Ohishi, Masako Kakizaki, Takashi Watanabe, Isao Oze, San-Lin You, Yumi Sugawara, Lesley M. Butler, Dong-Hyun Kim, Sue K. Park, Faruque Parvez, Shao-Yuan Chuang; Jin-Hu Fan; Chen-Yang Shen, Yu Chen, Eric J. Grant, Jung Eun Lee, Rashmi Sinha, Keitaro Matsuo, Mark Thornquist, Manami Inoue, Ziding Feng, Daehee Kang, John D. Potter

Corresponding author’s contact information:

Wei Zheng, M.D., Ph.D.

Vanderbilt Epidemiology Center

Vanderbilt University Medical Center

2525 West End Avenue, 8^th^ Floor

Nashville, TN 37203-1738

E-mail: [wei.zheng@vanderbilt.edu](mailto:wei.zheng@vanderbilt.edu)

**TABLE OF CONTENTS**

**PAGE 3-7 DESCRIPTIONS OF PARTICIPANTING COHORTS**

**DESCRIPTIONS OF PARTICIPANTING COHORTS**

**The Mumbai Cohort Study (Mumbai, India):** This prospective study was conducted with recruitment in two phases, between 1991-1994 and 1994-1996. At recruitment, cohort members were residents of Mumbai, India, 35 years of age and older. Approximately 150,000 subjects were interviewed for demographic, economic, and lifestyle variables. To ascertain vital status, an active house-to-house follow-up after an average 5.5 years was also conducted in two phases. Cause of death was obtained from the local death registry and linked to study participants using international classification of diseases 10th revision codes (ICD–10) [1].

**Trivandrum Oral Cancer Screening Trial (TOCS, India):** The Trivandrum Oral Cancer Screening trial is a cluster-randomised controlled trial, including apparently healthy subjects aged 35 years and older in 13 clusters randomized to either the intervention group (n=7) or the control group (n=6). The cohort is established since 1995 and still on-going. A total of 59,894 eligible subjects in the intervention group and 54,707 subjects in the control group were included at baseline. Subjects in the intervention group received 3 rounds of screening consisting of oral visual inspection by trained health workers at 3-year intervals. Subjects in the control group received messages on the harmful aspects of tobacco. Questionnaires were administered by health workers at each round in the 2 groups. Data on oral cancer incidence and mortality are continuously monitored by record linkage with the Trivandrum population-based cancer registry and municipal death registration systems [2].

**Health Effects of Arsenic Longitudinal Study (Bangladesh):** This cohort includes approximately 20,000 men and women. For each cohort member, active follow-up is through biennial in-person visits by trained study physicians who do detailed clinical and diagnostic work-ups for the detection of study outcomes.  This is supplemented by biannual in-person visits by village health workers and passive follow-up through study clinics dedicated exclusively to provide health care to the cohort members [3].

**China National Hypertension Survey Epidemiology Follow-up Study (CHEFS, China):** Baseline risk factors were carefully collected during the 1991 China National Hypertension Survey. A follow-up study was conducted during 1999-2000; it included in-person interviews to ascertain disease status and vital information for all study participants and a review of hospital records and death certificates for cardiovascular and renal events. The follow-up proportion was 95% over a period of 8 years. The CHEF study will provide unique and important information for developing national prevention strategies to reduce the societal burden of cardiovascular and renal disease in China [4].

**The Shanghai Cohort Study (SCS, China):** This was established in 1986. A total of 18,244 men aged 45-64, residing in four communities of Shanghai, China were recruited to the study. At recruitment, dietary and medical histories, as well as blood and urine specimens, were collected. The most recent, complete follow-up was conducted in 2008. The local cancer registry, vital statistics unit, and annual in-person visits are used to identify cancer occurrence, death, and other major health outcomes, including cardiovascular disease [5].

**The Shanghai Men’s Health Study (SMHS, China):** This population-based cohort study includes 61,504 Chinese men aged 40-74, free of cancer at enrollment, and living in seven communities in urban Shanghai, China. Recruitment for the SMHS was initiated in April 2002 and completed in June 2006. Detailed information on dietary and other lifestyle factors was collected at baseline and is being updated with a follow-up survey using a validated questionnaire. At baseline, peripheral blood samples from 46,354 participants, and urine samples from 54,807 participants, were collected. Participants, who did not donate a blood sample, donated a buccal cell sample [6].

**The Shanghai Women’s Health Study (SWHS, China):** This population-based cohort was established during 1997-2000. A total of 74,942 women, aged 40-70, in Shanghai were recruited; these cohort members have been followed up with a combination of record linkage and biennial interviews. Biologic samples were collected from 87.5% of the cohort. The most recent follow-up was completed in 2008. Deaths and cancer incidence are verified through the local cancer registry, vital-statistics unit, and in-person visits to cohort members [7].

**The Community-Based Cancer Screening Project (CBCSP, Taiwan):** This project was established in 1991. The study includes 23,820 male and female subjects aged 30-65 from seven townships in Taiwan, with a mean of 15.2 years of follow-up. Follow-up includes structured questionnaires administered by trained staff and computerized linkage of cancer-registration and death-certification systems to ascertain cancer incidence and death [8].

**CardioVascular Disease risk FACtor Two-township Study (CVDFACTS, Taiwan):** The study recruited participants from 1990-1993. In all, 5160 men and women, aged 18 and above, were recruited from Chu-Don, a Hakka community in northwest Taiwan and from Pu-Tze, a Fukien community in southern Taiwan. Five villages in each of the two townships were selected randomly from those with greater than 1000 people or with a population density greater than 200 per square kilometer. Follow-up is every 3 years. Death-certificate data are obtained from the Department of Health in Taiwan [9].

**The Singapore Chinese Health Study (SCHS, Singapore):** The Singapore Chinese Health Study is a long-term, population-based cohort study focused on the role of diet and its interplay with genetic factors in cancer etiology. Established in 1993, the study recruited a total of 63,257 men and women, aged 45-74. Upon recruitment, subjects were interviewed using a staff-administered questionnaire, which included a validated Food Frequency Questionnaire. Follow-up has included additional interviews and the collection of biologic samples for all consenting cohort members. The study conducts regular record linkages with local cancer and vital status registries [10].

**Three Prefecture Cohort Study Aichi (3 Pref Aichi, Japan):** This cohort study conducted a baseline survey with self-administered questionnaire regarding lifestyle on the residents aged 40 years or older in both urban area (Chikusa-ku in Nagoya City) and rural area (Inuyama Area) in Aichi Prefecture between 1983 and 1985. A total of 15,738 male (10,044 from urban area) and 17,777 female (11,740 from urban area) without having history of cardiovascular diseases or respiratory diseases were selected for the study subjects. The study subjects were followed up by linking to death certificate. The total observed person-years was 127,409 for male and 149,978 for female [11].

**Ibaraki Prefectural Health Study (Ibaraki, Japan):**  A total of 33,336 men and 64,272 women, 40 to 79 years of age, with no history of stroke or coronary heart disease, completed a baseline risk factor survey in 1993 under the auspices of the Ibaraki Prefectural Health Study. Systematic mortality surveillance was performed through 2003, and 264 intra-parenchymal hemorrhage deaths were identified. Participants are followed up every 2 years and deaths are certified through systematic review of death certificates and resident registrations [12].

**Japan Collaborative Cohort Study (JACC, Japan):** The study has contributions from 45 areas of the country and now covers 86,682 participants, enrolled at various centers located from Hokkaido in the North to Kyushu in the South. To collect epidemiologic information at baseline, a self-administered questionnaire was used. Follow-up is current to 2003 in the majority of cases and a total of 12,888 deaths were registered; the five most common sites of cancer development are lung, stomach, liver, pancreas, and colon in men, and the stomach, lung, pancreas, colon and liver in women [13].

**The Japan Public Health Center-based Prospective Study (JPHC1 AND JPHC2, Japan):** The first cohort was established in 1990, followed by a second cohort in 1993. A total of 140,420 men and women, aged 40-59 were recruited for the study. Self-administered questionnaires and biologic samples were collected at baseline, and participants were followed up with additional surveys after five and ten years. Incidence of cancer and cardiovascular diseases, as well as information on death and migration, are captured annually [14].

**Three Prefecture Cohort Study Miyagi (3 Pref Miyagi, Japan):** This cohort study conducted a baseline survey with two self-administered questionnaires, regarding lifestyle and personality, on the residents aged 40 to 64 years, in 14 municipalities of Miyagi Prefecture, Japan, during June through August, 1990. Out of the eligible 51,921 residents, 47,605 (91.7%) responded to the lifestyle questionnaire and form the cohort under study. This includes 13,992 men and 17,353 women. The list of study subjects in the cohort has been linked with the Miyagi Prefectural Cancer Registry data through December 31, 1997 [15].

**The Miyagi Cohort Study (Miyagi, Japan):** This population-based cohort was established in 1990. Subjects were residents of fourteen municipalities in the Miyagi Prefecture aged 40-64. In total, 47,605 men and women were recruited to the study. Follow-up includes linkage with the local cancer registry and death certificates [16].

**The Ohsaki National Health Insurance Cohort Study (Ohsaki, Japan):** This cohort was established in 1995. Subjects were National Health Insurance beneficiaries, aged 40-79, who received care at the Ohsaki Public Health Center. A total of 51,253 male and female subjects were recruited. The last follow-up was completed in December 2003. The study continues to conduct monthly reviews of death certificates and National Health Insurance files. Cancer incidence is obtained from the local cancer registry [17].

**Radiation Effects Research Foundation (RERF, Japan):** The Life Span Study of atomic bomb survivors follows a cohort of 120,000 persons, 94,000 of whom were in the city at the time of the bombing and another 26,000 age- and sex-matched residents who were not in Hiroshima or Nagasaki at the time of the bombing. The cohort was established based on the 1950 Japanese national census and continues to be followed for mortality and cancer incidence. Lifestyle information was collected via a clinical sub-study and mailed questionnaires. Data used for this study were restricted to those who were exposed to less than 0.1 gray of bomb radiation [18].

**The Korea Multi-center Cancer Cohort (KMCC, Republic of Korea):** During the period 1993-2004, 19,688 men and women over 18 years of age were recruited from four areas of Republic of Korea. Interviews at the time of enrollment collected information on past histories, lifestyle factors, physical activity, reproductive factors, and in-brief diet and agricultural exposures. Biologic samples have been collected for a subset of the cohort. Follow-up includes record linkage with the national cancer registry, death-certificate system, and health-insurance databases [19].

**The Seoul Male Cohort Study (SMCS, Republic of Korea):** The cohort was established in 1992. A total of 29,918 men, aged 40-59 were recruited for the study. Self-administered questionnaires were collected at baseline from 14,533 participants. Cohort participants were followed from January 1, 1993 to December 31, 2008. Mortality data were collected annually using death certificates from the National Statistics Office. [20].

**References for supplementary methods**

[1] Pednekar MS, Hakama M, Hebert JR, Gupta PC (2008) Association of body mass index with all-cause and cause-specific mortality: findings from a prospective cohort study in Mumbai (Bombay), India. Int J Epidemiol 37: 524-35.

[2] Sankaranarayanan R, Ramadas K, Thomas G, Muwonge R, Thara S, Mathew B, Rajan B and for the Trivandrum Oral Cancer Screening Study Group. (2005). Effect of screening on oral cancer mortality in Kerala, India: a cluster-randomised controlled trial. Lancet 365:1927–1933

[3] Ahsan H, Chen Y, Parvez F, Hussain AZMI, Momotai H, et al. (2006) Health Effects of Arsenic Longitudinal Study (HEALS):  Description of a Multidisciplinary Epidemiologic Investigation.  J Exp Sci Environ Epidemiol 16: 191-205.

[4] He J, Gu D, Chen J, Wu X, Kelly TN, et al. (2009) Premature deaths attributable to blood pressure in China: a prospective cohort study. Lancet 374: 1765-72.

[5] Moy KA, Yuan J-M, Chung F-L, Wang X-L, Van Den Berg D, et al. (2009) Isothiocyanates, glutathione S-transferase M1 and T1 polymorphisms and gastric cancer risk: a prospective study of men in Shanghai, China. Int J Cancer 125: 2652-9.

[6] Cai H, Yang G, Xiang YB, Hebert J, Liu D, et al. (2005) Sources of variation in nutrient intakes among men in Shanghai, China. Public Health Nutr 8: 1293-9.

[7] Zheng W, Chow WH, Yang G, Fan J, Rothman N, et al. (2005) The Shanghai Women’s Health Study: rationale, study design, and baseline characteristics. Am J Epidemiol 162: 1123-31.

[8] Chen CJ, You SL, Lin YP, CBCSP Study Group (1993) Community-based cancer screening project (CBCSP) in Taiwan. In: Chin BC, Yao C (eds.) Proceedings of 1993 Chinese American Academic and Professional Convention. Chinese American Academic and Professional Society, Oak Brook, pp. 937-40.

[9] Chuang SY, Bai CH, Chen WH, Lien LM, Pan WH (2009) Fibrinogen independently predicts the development of ischemic stroke in a Taiwanese population: CVDFACTS study. Stroke 40: 1578-84.

[10] Hankin JH, Stram DO, Arakawa K, Park S, Low SH, et al. (2001) Singapore Chinese Health Study: development, validation, and calibration of the quantitative food frequency questionnaire. Nutr Cancer 39: 187-95.

[11] Marugame T, Sobue T, Satoh H, Komatsu S, Nishino Y, et al. (2005) Lung cancer death rates by smoking status: comparison of the Three-Prefecture Cohort study in Japan to the Cancer Prevention Study II in the USA. Cancer Sci 96: 120-6.

[12] Matsuo T, Sairenchi T, Iso H, Irie F, Tanaka K, et al. (2008) Age- and gender-specific BMI in terms of the lowest mortality in Japanese general population. Obesity 16: 2348-55.

[13] Tamakoshi A, Yoshimura T, Inaba Y, Ito Y, Watanabe Y, et al. (2005). Profile of the JACC study. J Epidemiol 15 Suppl 1:S4-8.

[14] Tsugane S, Sobue T (2001) Baseline survey of JPHC study—design and participation rate. Japan Public Health Center-based Prospective Study on Cancer and Cardiovascular Diseases. J Epidemiol 11: S24-9.

[15] Nishino Y, Suzuki Y, Ohmori K, Hozawa A, Ogawa K, Kuriyama S, Tsubono Y, Shibuya D, Tsuji I, Fukao A, Hisamichi S. (2004) Cancer incidence profiles in the Miyagi Cohort Study. J Epidemiol. 14 Suppl 1:S7-11

[16] Tsuji I, Nishino Y, Tsubono Y, Suzuki Y, Hozawa A, et al. (2004) Follow-up and mortality profiles in the Miyagi Cohort Study. J Epidemiol 14 Suppl 1:S2-6.

[17] Tsuji I, Nishino Y, Ohkubo T, Kuwahara A, Ogawa K, et al. (1998) A prospective cohort study on National Health Insurance beneficiaries in Ohsaki, Miyagi Prefecture, Japan: study design, profiles of the subjects and medical cost during the first year. J Epidemiol 8: 258-63.

[18] Ozasa K, Shimizu Y, Suyama A, Kasagi F, Soda M, Grant EJ, Sakata R, Sugiyama H, Kodama K. (2012) Studies of the Mortality of Atomic Bomb Survivors, Report 14, 1950-2003: An Overview of Cancer and Noncancer Diseases. Radiation Research 177:229–43.

[19] Cho LY, Kim CS, Li L, Yang JJ, Park B, et al. (2009) Validation of self-reported cancer incidence at follow-up in a prospective cohort study. Ann Epidemiol 19: 644-6.

[20] Rhee CW, Kim JY, Park BJ, Li ZM, Ahn YO. (2012) Impact of individual and combined health behaviors on all causes of premature mortality among middle aged men in Korea: the Seoul Male Cohort Study. J Prev Med Public Health. 45(1):14-20
